# Severe Osteoporosis in a Young Woman With Type 1 Diabetes and Pancreatic Exocrine Insufficiency

**DOI:** 10.1155/crie/3988709

**Published:** 2026-06-16

**Authors:** Jeremy A. Knott, Terrence H. Diamond

**Affiliations:** ^1^ Department of Endocrinology, St George Hospital, Sydney, New South Wales, Australia, nsw.gov.au; ^2^ Faculty of Medicine, The University of New South Wales Sydney, Sydney, New South Wales, Australia, unsw.edu.au

**Keywords:** bone health in diabetes, osteoporosis, pancreatic exocrine insufficiency, type 1 diabetes

## Abstract

Patients with type 1 diabetes mellitus (T1DM) are at increased risk of osteoporosis; however, contributing secondary factors may be under‐recognised. We report a case of a 29‐year‐old woman with a 10‐year history of suboptimally controlled T1DM (HbA1c 10.1%–14.7%) who presented with an atraumatic tibial fracture and vertebral compression fractures. She had a history of significant weight loss (nadir 37 kg) and chronic malabsorption. Investigations demonstrated severe pancreatic exocrine insufficiency (PEI; faecal elastase 42 μg/g), vitamin D deficiency (25 nmol/L), and secondary hyperparathyroidism (PTH 10.8 pmol/L). Dual‐energy X‐ray absorptiometry revealed reduced bone mineral density at the total hip (*Z*‐score –3.4) and lumbar spine (*Z*‐score –2.1). Management included pancreatic enzyme replacement, vitamin D and calcium repletion, and optimisation of glycaemic control. At the 12‐month follow‐up, biochemical parameters improved (vitamin D 112 nmol/L), and no further fractures occurred. This case highlights the importance of identifying secondary contributors to bone fragility in young patients with T1DM, particularly malabsorption due to PEI.

## 1. Introduction

The prevalence of osteoporosis is increased in patients with type 1 diabetes mellitus (T1DM), with meta‐analyses reporting up to a 6.3‐fold greater relative risk of hip fractures compared to patients without diabetes [1, 2]. Despite this, the skeletal complications of diabetes are often under‐recognised, and the fracture risk assessment and management remain challenging. The pathophysiology of bone fragility in patients with T1DM is multifactorial, attributed to low bone mass and decreased bone formation due to insulin and insulin‐like growth factor deficiencies, which are considered osteoanabolic [2, 3]. Chronic hyperglycaemia further contributes through accumulation of advanced glycation end‐products in bone collagen, microangiopathy and increased marrow adiposity [4]. The association of T1DM with autoimmune malabsorptive conditions, such as coeliac disease, is well recognised [5]. Pancreatic exocrine insufficiency (PEI) is increasingly recognised in T1DM [6] and may result in clinically significant malabsorption, including vitamin D [7] and calcium deficiency, which may exacerbate skeletal fragility. Despite this, there are limited case reports describing severe bone disease in patients with coexisting T1DM and PEI, highlighting the need to consider less common secondary contributors to bone fragility in this population.

## 2. Case Report

**Figure 1:**
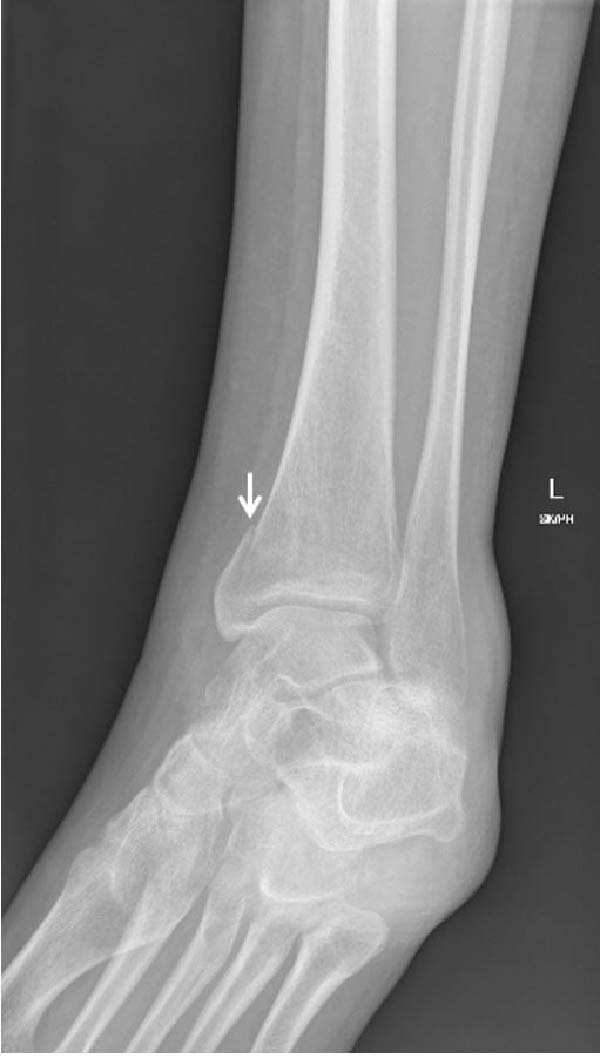
A non‐displaced fracture (arrow) of the left lower tibia on frontal ankle X‐ray.

A 29‐year‐old female with a 10‐year history of suboptimally controlled T1DM (Hba1c 10.1%–14.7%; reference: <6.5%) presented with an atraumatic left tibial fracture (Figure 1), prompting further investigation. Over the preceding years, she has developed significant weight loss of 25 kg (nadir weight of 37 kg) in the context of chronic malabsorption due to previously unrecognised severe PEI, associated with prolonged vitamin D deficiency. During this period, she also developed a 4‐year history of amenorrhoea secondary to weight‐related hypothalamic hypogonadism, with documented low gonadotrophins (FSH <1 IU/L; reference: 3.9–8.8 IU/L follicular phase, LH <0.1 IU/L; reference: 2.9–10.1 IU/L follicular phase) and low oestradiol <70 pmol/L (reference: 100–450 pmol/L follicular phase). Her diabetes was complicated by proliferative retinopathy, polyneuropathy, nephropathy (urine albumin:creatinine ratio 534 mg/mmol; reference: < 3.5 mg/mmol) and autonomic neuropathy with gastroparesis and postural hypotension (erect blood pressure decline of 20 mmHg).

At the time of review, she had regained her weight and was receiving a combined oral contraceptive pill (levonorgestrel/ethinylestradiol), providing exogenous oestrogen exposure in the setting of her previously prolonged hypothalamic amenorrhoea. She denied steatorrhoea or loose stools but had persistent biochemical evidence of malabsorption. She did not smoke or consume alcohol.

**Figure 2:**
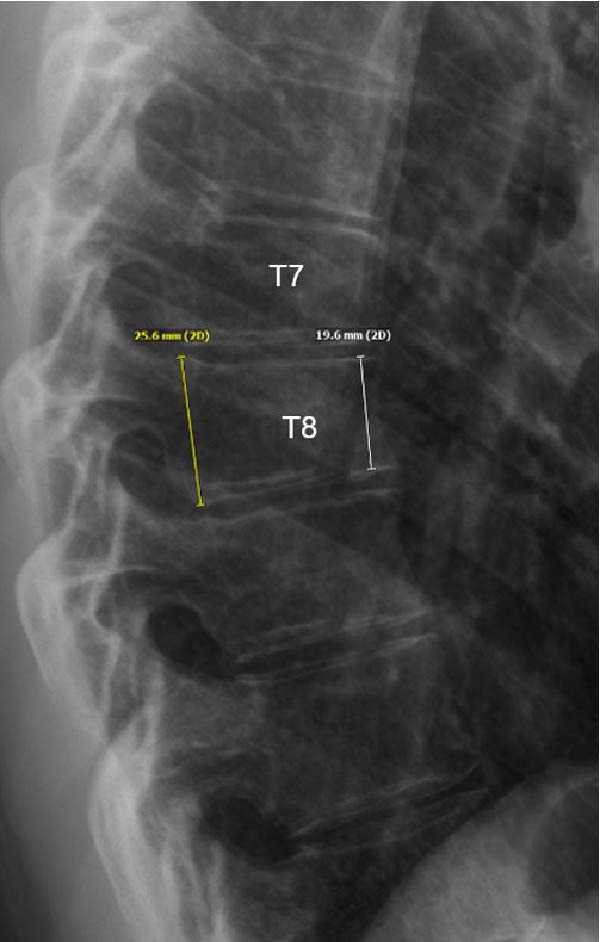
Lateral projection X‐ray of the thoracolumbar spine demonstrated >20% anterior compression of both T7 and T8 vertebral bodies, with T8 outlined for reference.

Physical examination revealed sustained left ankle swelling as well as reduced muscle strength. Body composition assessment using bioelectrical impedance analysis confirmed a low skeletal muscle mass of 19.2 kg with an elevated fat mass of 25.5 kg, relative to her total body weight of 62.4 kg with a normal body mass index of 22.9 kg/m^2^ (reference: 18.5–24.9 kg/m^2^). Spinal X‐rays demonstrated >20% anterior compression fractures of T7 and T8 vertebral bodies (Figure 2). A dual‐energy X‐ray absorptiometry scan demonstrated significantly reduced total hip bone mineral density of 0.60 g/cm^2^ (*Z*‐score of −3.4). Her lumbar spine (L2‐L4) bone mineral density was 0.95 g/cm^2^ (*Z*‐score of −2.1).

Laboratory investigations revealed chronic malabsorption with a low serum 25‐vitamin D of 15 nmol/L (reference: 50–250 nmol/L), ferritin of 8 μmol/L (reference: 30–310 μmol/L), vitamin B12 of 12 pmol/L (reference: 140–670 pmol/L) as well as PEI with a low faecal elastase of 42 mcg/g (reference: > 200 mcg/g). She had an ionised serum calcium of 1.19 mmol/L at the lower limit of normal (reference: 1.15–1.33 mmol/L), normal total serum calcium of 2.14 mmol/L (reference: 2.1–2.6 mmol/L), secondary hyperparathyroidism with an elevated PTH of 10.8 pmol/L (reference: 1.6–6.9 pmol/L) and a low 24 h urinary calcium excretion of 0.6 mmol/24 h (reference: 2.5–7.5 mmol/24 h). Coeliac serology and cystic fibrosis transmembrane conductance regulator genetic testing were negative, excluding coeliac disease and cystic fibrosis as contributing factors to her chronic malabsorption. Her bone turnover markers were at the upper end of normal with a P1NP of 88 mcg/L (reference: 15–90 mcg/L) and a CTx of 560 mcg/L (reference: 150–800 mcg/L). She had an elevated alkaline phosphatase of 117 U/L (reference: 20–105 U/L) and her phosphate was normal 0.94 mmol/L (reference: 0.75–1.5 mmol/L). Prior abdominal magnetic resonance enterography performed for investigation of her chronic weight loss demonstrated no intra‐abdominal or pancreatic structural abnormalities.

Therapy was initiated with oral cholecalciferol 5000 IU daily, calcium citrate 250 mg TDS and pancrelipase enzyme capsules 75,000 IU TDS. Intensive diabetic management was achieved using a Medtronic‐770G closed‐loop insulin pump and dietary adjustments. A multidisciplinary diabetes team was essential in improving her glycaemia and reducing her hypoglycaemia risk, which was increased due to her hypoglycaemia unawareness from autonomic neuropathy and combined insulin‐glucagon deficiency from her pancreatic endocrine/exocrine insufficiency. A muscle‐strengthening physiotherapy program was implemented to reduce her fall risk. Parenteral bisphosphonate therapy was considered but not commenced, as management was prioritised towards correction of metabolic abnormalities and her reproductive potential. At the 12‐month follow‐up, supplementation and pancreatic enzyme replacement were associated with improvement in biochemical parameters (vitamin D 112 nmol/L), consistent with improved mineral metabolism. The patient remained fracture‐free with no new skeletal events. Interval bone mineral density assessment at 6 months demonstrated overall stability, with small increases in lumbar spine BMD (0.95–0.97 g/cm^2^) and total hip BMD (0.60–0.61 g/cm^2^).

## 3. Discussion

Long‐term exposure to a hyperglycaemic environment with microvascular disease may affect bone microvasculature and bone marrow microenvironment, where bone progenitor cells reside. This can cause a shift in production towards adipocytes, away from osteoblasts, resulting in an increase in bone marrow adiposity [8]. These changes predispose bone to increased fracture risk and impaired osseous healing. In addition, microvascular complications, including neuropathy, retinopathy and postural orthostatic hypotension, increase fall risk, which may also partially account for increased fracture risk [9], which was an important consideration in our patient’s ongoing management. It has been shown that intensive insulin therapy can contribute to the stabilisation of bone mass and reduction in bone resorptive markers in patients with T1DM by restoring anabolic activity [10].

Furthermore, PEI can often occur in patients with T1DM [11]. Proposed pathophysiological mechanisms include acinar atrophy and fibrosis due to autoimmune and inflammatory‐mediated injury as well as a progressive decrease in pancreatic volume, resulting in a clinically significant reduction of digestive exocrine enzymes [11]. Accordingly, faecal‐elastase screening should be considered in patients with T1DM with gastrointestinal symptoms to optimise exocrine function [11] and ultimately bone health, as patients with PEI have been shown to demonstrate a high prevalence of osteoporosis [12]. Pancreatic enzyme supplementation has also been shown to reduce osteopathy in patients with PEI [12].

Our patient demonstrated severe vitamin D deficiency with secondary hyperparathyroidism, low urinary calcium excretion, and elevated alkaline phosphatase, consistent with impaired mineral metabolism contributing to bone fragility. Although a bone biopsy was not performed, there were no classical clinical features such as bone pain or tenderness, nor radiological features such as Looser’s zones or trabecular blurring to suggest a diagnosis of osteomalacia [13]. Secondary hyperparathyroidism further exacerbates bone loss through increased bone turnover, cortical thinning and mineralisation defects [13, 14], while vitamin D and calcium repletion are known to improve biochemical parameters and increase bone density [15, 16]. In addition, her previous weight‐related hypogonadism with sustained hypoestrogenism likely compounded bone fragility, as oestrogen deficiency promotes increased bone resorption and deterioration of trabecular microarchitecture [17]. Exogenous oestrogen exposure would be expected to attenuate oestrogen‐deficient bone loss, with physiologic oestrogen replacement shown to improve bone mineral density in women with hypothalamic oligo‐amenorrhoea [18].

Currently, there are no specific therapeutic guidelines for osteoporosis management in patients with T1DM. Available evidence suggests that diabetes does not substantially alter the efficacy of anti‐osteoporotic therapies. A systematic review evaluating risedronate, alendronate, raloxifene and teriparatide in patients with and without diabetes reported comparable gains in bone density and reductions in vertebral fracture risk across both groups [19]. In our patient, the coexistence of T1DM with PEI and vitamin D deficiency and secondary hyperparathyroidism emphasises that correction of malabsorption, vitamin D and calcium repletion, and optimisation of glycaemic control are essential prerequisites before anti‐osteoporotic therapy is considered, particularly given her young age and reproductive potential. Anti‐resorptive therapies, including bisphosphonates and denosumab, are effective options for fracture risk reduction; however, in this case, their use was deferred in the setting of untreated secondary contributors to bone disease and the need for long‐term therapy. In particular, denosumab requires ongoing administration, with rapid bone loss following cessation, which is an important consideration to avoid in younger patients [20].

Animal studies have demonstrated that intermittent parenteral parathyroid hormone analogues, such as abaloparatide and teriparatide, can restore bone mass in diabetic models through enhanced osteoblast activity [21]. However, their clinical role is limited in the presence of secondary causes of osteoporosis, such as vitamin D‐deficient secondary hyperparathyroidism, evident in this case. As sclerostin, a negative regulator of bone formation, is upregulated in diabetes, inhibitors of this pathway are under investigation and may eventually expand treatment options [22]. However, our case highlights that management must first prioritise correction of secondary metabolic abnormalities and stabilisation of bone health before novel agents can be meaningfully applied.

## 4. Conclusion

Our case highlights the complex nature of osteofragility fractures in a young individual with T1DM and the need for comprehensive investigation of potential contributing factors. Continual follow‐up and coordination among specialists are vital to ensure the patient’s long‐term skeletal health and overall well‐being. Further research is warranted to better understand the presentation, early detection and mechanism of osteofragility fractures in T1DM‐related PEI.

## 5. Learning Points

1. Patients with T1DM are at increased risk of developing severe osteoporosis due to reduced bone mineralisation attributed to insulin deficiency, chronic hyperglycaemia and autoimmune inflammation necessitating regular bone health assessments.
2. Exocrine pancreatic insufficiency and other autoimmune malabsorptive conditions are important contributors to bone fragility in patients with T1DM, predisposing to vitamin D deficiency and secondary hyperparathyroidism, which should be considered.
3. Management of diabetes‐related osteofragility fractures requires a multifactorial approach for correction of all metabolic abnormalities and early identification of contributing co‐morbidities.

## Author Contributions

**Jeremy A. Knott**: writing – original draft. **Terrence H. Diamond:** writing – review and editing.

## Funding

No funding was received for this manuscript.

## Disclosure

Our case report was presented as a conference poster presentation at the Australian and New Zealand Bone and Mineral Society in 2023, which can be viewed at https://anzbms-2023.p.asnevents.com.au/days/2023-10-23/abstract/97281 [23].

## Consent

Written informed consent was obtained from the patient to publish this report in accordance with the journal’s patient consent policy.

## Conflicts of Interest

The authors declare no conflicts of interest.

## Data Availability

The data that support the findings of this study are available from the corresponding author upon reasonable request.
